# Minimizing leukemia escape: implementing a dual anti-CD20- and CD19-scFv-based chimeric antigen receptor (CAR)

**DOI:** 10.1186/2051-1426-3-S2-P122

**Published:** 2015-11-04

**Authors:** Dina Schneider, Ying Xiong, Andre Roy, Andrew Kaiser, Boro Dropulic, Rimas Orentas

**Affiliations:** 1 Lentigen Technology Inc., Gaithersburg, MD, USA; 2 Miltenyi Biotec GmbH, Bergisch Gladbach, Germany

## 

Adoptive immunotherapy with chimeric antigen receptor (CAR) transduced T lymphocytes has shown promising results in both pediatric and adult B cell malignancies. Nevertheless, both CAR-based and antibody-based anti-CD19 therapies, *e.g.* blinatumomab, have seen treatment failures attributed to the loss of CD19 or an epitope of CD19 on the surface of the malignant B cell. It may be possible to overcome antigen escape by targeting two tumor antigens simultaneously, *i.e.* CD19 and CD20 using a tandem construct with two scFv-based CAR binding domains. Lentiviral vectors encoding chimeric antigen receptors comprised of anti-CD19 and anti-CD20 targeting domains expressed alone or in tandem were transduced into T cells from healthy donors to generate the corresponding CAR19, CAR20, CAR19_20 (CD19 scFv more distal to the T cell plasma membrane) and CAR20_19 T cells (CD20 scFv distal). The transduced T cells were 50-70% CAR positive as determined by protein L flow cytometric analysis. Expression of CAR proteins of the expected molecular weight was confirmed by Western blot analysis of transduced T cells. When CAR-transduced T cells were combined with CD19^+^CD20^+^ Raji target cells, but not CD19^-^CD20^-^ K562 cells, all four CAR T cell types demonstrated comparable efficient killing of leukemia targets (E:T ratio >2), and target-dependent induction of IFN-γ, as measured in co-culture supernatants by ELISA.

We then began a series of *in vitro* co-culture experiments where we used very low E:T ratios to examine the potential for CAR-induced antigen loss on surviving leukemia cells. All CAR-T cells expressing an anti-CD19 scFv induced rapid loss of CD19 surface expression. In contrast, the CD20 surface marker was less prone to down-regulation by CAR-T cells expressing anti-CD20 scFv. Upon flow cytometric analysis of surviving leukemia cells on day 5, CD19 expression was reduced to 3%, 48%, 73%, 90% and 93% of control when co-cultured with CAR T cells expressing CAR19_20, CAR20_19, CAR19, CAR20, and control T cells, respectively. Similar results were seen when experiments were of longer, 7 days, or shorter, 1 day, duration. In conclusion, tandem CAR T cells are as effective as single CAR19 or CAR20 T cells in leukemia cell killing. Importantly, dual scFv-expressing CARs are more potent in preventing tumor antigen escape via target antigen down-regulation.

